# Impact of Agronomic Treatments on the Enzymatic Browning of Eggplants (*Solanum melongena* L.)

**DOI:** 10.3390/antiox12020410

**Published:** 2023-02-08

**Authors:** Peyman Ebrahimi, Carlo Nicoletto, Paolo Sambo, Federica Tinello, Dasha Mihaylova, Anna Lante

**Affiliations:** 1 Department of Agronomy, Food, Natural Resources, Animals, and Environment—DAFNAE, Agripolis, University of Padova, 35020 Legnaro, Italy; 2 Department of Biotechnology, University of Food Technologies, 26 Maritza Blvd., 4002 Plovdiv, Bulgaria

**Keywords:** polyphenol oxidase (PPO), reactive oxygen species (ROS), antioxidants, polyphenols, colorimetry, oxidation, fertilizer, grafting

## Abstract

Enzymatic browning could negatively affect the sensory and nutritional properties of eggplants post-harvest. Polyphenols, polyphenol oxidase (PPO), and reactive oxygen species (ROS) are three material conditions involved in enzymatic browning. This paper seeks to evaluate the effect of fertilization techniques and grafting on the activity of PPO and colorimetric parameters in cultivated eggplants. Fertilization alone significantly increased the PPO activity in all eggplant fleshes (*p* ≤ 0.05), whereas the grafting technique combined with fertilization decreased the PPO activity in most of the samples significantly (*p* ≤ 0.05). Moreover, there was a significant positive correlation between the PPO activity and the a* values of the eggplants. The a* values in grafted eggplants were significantly different from each other (*p* ≤ 0.05), showing that grafting the fertilized eggplants could be effective in controlling the enzymatic browning. The eggplant slices exposed to air for 60 min at room temperature showed a significant increase (*p* ≤ 0.05) in PPO activity, browning index (BI), total color difference (ΔE), and a*, b*, and c* values. Thus, it is necessary to minimize the exposure time of the slices to air at room temperature, even if combining fertilization techniques with grafting could delay the enzymatic browning in fresh-cut eggplants.

## 1. Introduction

The eggplant (*Solanum melongena* L.) belongs to the family Solanaceae and is the fifth most popular vegetable, with an approximate annual production of 56.6 million tons worldwide. Eggplant is considered a low-calorie functional food because it is a valuable source of phenolic compounds, dietary fibers, proteins, vitamins, and minerals [1,2]. The high phenolic content in edible plants such as eggplant could inhibit or delay the oxidation process arising from reactive oxygen species (ROS), which can consequently result in decreased oxidative damage in food products [2,3,4]. However, eggplant deteriorates rapidly after harvesting. It has a limited shelf life at ambient temperature as it is susceptible to browning, flavor loss, softening, and infectious diseases [5]. The development of browning may be responsible for wasting more than 50% of food products [6]. Therefore, identifying more optimal procedures to prevent the post-harvest problems of the eggplant, such as browning, seems to be an open question.

Enzymatic browning is a common phenomenon resulting from the mechanical and physical stresses that occur in the harvesting, storage, transportation, and processing of fruits. It is considered a negative sensory attribute in eggplants and products containing eggplants [1,7,8]. Substrates (phenolic compounds), enzymes, and ROS are three material conditions for enzymatic browning [9]. Although phenolic compounds play an important role as natural antioxidants and nutritional substances in plant-based foods, they can act as a substrate for polyphenol oxidase (PPO) and increase the risk of browning in plants. Since the activity of PPO can decrease the content of polyphenols, it can lead to an increase in ROS [4,10], resulting in the acceleration of the senescence process of the plant [11]. However, the major factor governing browning is the activity of the PPO enzyme, an intracellular *o*-diphenol oxidase [1,12].

PPO is a copper-containing enzyme belonging to the oxidoreductase family. It can trigger the oxidation of *o*-diphenols and changes them into highly reactive *o*-quinones [4]. Quinones are electrophilic molecules that can covalently react with nucleophiles, such as phenolic compounds, amines, and thiols, resulting in the generation of black or dark brown substances (i.e., melanin) [13]. This reaction causes a color change and decreases the antioxidant activity in food products, leading to the deterioration of the nutritional properties of food matrices. Therefore, applying a suitable inhibitory method before or after the harvest of the plant to decrease the disadvantages derived from PPO activity is crucial [10]. 

Partial or full inhibition of PPO could be implemented by removing the enzyme substrates (e.g., phenolic compounds), controlling the pH of the medium, adding sodium sulfite or ascorbic acid, or through thermal inactivation [14]. Fertilization also could impact the PPO activity and phenolic expression of plants [15]. Since there is a significant challenge in reducing the use of synthetic fertilizers, the utilization of organic fertilizers could be an alternative solution to increase the sustainability of agricultural procedures [16]. In addition, grafting is an agronomic technique used to improve the characteristics of crops, such as tolerance to pathogens and abiotic stress, plant survival, apparent fruit quality, and health-related components [17,18]. Therefore, the impact of grafting and fertilization on the quality of plants is not fully understood.

To our knowledge, there is no research investigating the effects of fertilization and grafting on the enzymatic browning of eggplants. However, in managing the shelf-life of eggplants and the waste of products after harvesting, fresh-cut technology plays a strategic role because it is considered a mandatory step in preparing semi-finished products that are ready to cook or for freezing. Therefore, this paper aims to evaluate the effect of the agronomical treatments and fresh-cut technology on the post-harvest enzymatic browning that impacts the antioxidant potential of fresh-cut eggplants.

## 2. Materials and Methods

### 2.1. Chemicals and Materials

Eggplant seedlings (*Solanum melongena* L. cv. SV2162EV) were purchased from Seminis (Milan, Italy). Bovine serum albumin (BSA; 2 mg protein/mL), catechol, citric acid, Coomassie Plus Protein Assay Reagent, polyvinylpolypyrrolidone (PVPP), trisodium citrate dihydrate, and Triton X-100 were purchased from Sigma-Aldrich (St. Louis, MO, USA). The water utilized in all analyses was deionized and distilled.

### 2.2. Cultivation of Eggplants

**Figure 1:**
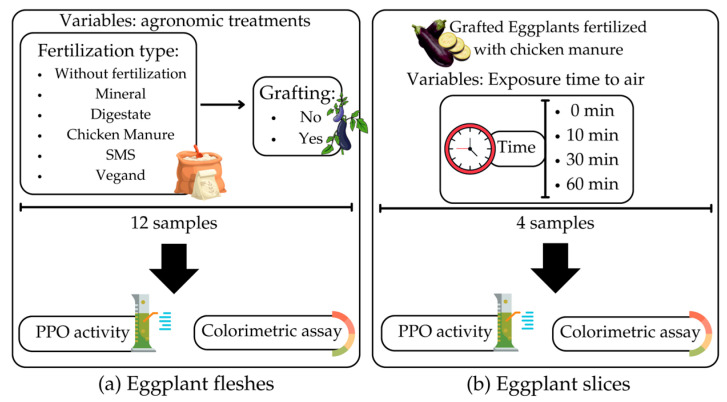
Experimental design and sample preparation methods in eggplant fleshes (**a**) and eggplant slices (**b**). PPO: polyphenol oxidase; SMS: spent mushroom compost.

Eggplants were cultivated in triplicate at the experimental farm of the University of Padova, Legnaro, Italy. The cultivation was performed in mulched soil with a polyethylene plastic film, and a micro-flow irrigation system was set up for drip irrigation. The plants were grown in the soil inside a greenhouse tunnel (area: 400 m^2^, 8 × 50 m) with a plastic-covered roof. The planting density was the distance between the plants: 50 cm on the row and 1 m between the rows. Biological control methods were used for crop protection by exploiting insects against aphids, thrips, and mites. The experimental plan was a split-plot scheme. Different fertilizers, including mineral, chicken manure, digestate, spent mushroom compost (SMS), and Vegand^®^, were considered in the plots (66 m^2^ each). Grafted/not-grafted plants were in the subplots. Each plot was compared with an unfertilized control. The grown plants were grafted using dynafort rootstock (Seminis, Milan, Italy), a strong rootstock variety with fast germination, long leg length, and high grafting potential. Therefore, 12 samples were cultivated, as shown in Figure 1a, with approximately the same pH, dry matter, titratable acidity, and brix (data not shown).

### 2.3. Sample Preparation

After harvesting, the samples were manually peeled, and the fleshes of the fruits were cut into small pieces (2–4 cm) to investigate the effect of the agronomic treatments. In another group (Figure 1b), grafted eggplants fertilized with chicken manure were cut into slices 1 cm thick to evaluate the effect of exposure to air at different times (0, 10, 30, and 60 min). The fleshes and slices were subjected to colorimetric analysis immediately after cutting. Before the PPO activity assay, the eggplants were lyophilized under a pressure of 28 mbar at −50 °C using a freeze dryer (Modulyo, Edwards, West Sussex, UK), and the lyophilized samples were ground in a laboratory miller (M20, IKA-Werke GmbH and Co. KG, Staufen, Germany). The dried, ground powder was passed through a standard mesh number 20 (particle size < 0.850 mm). The homogenized, freeze-dried ground material was mixed well and stored at −18 °C until the extraction of the PPO.

### 2.4. Extraction of PPO

The extraction of PPO was performed in triplicate following the method developed by Tinello et al. (2018), with some modifications [19]. First, 0.1 g of freeze-dried eggplant powder was added to 10 mL of sodium citrate buffer (0.1 M, pH 6.0) containing 0.1% (*w*/*v*) Triton X-100 and 0.5 g of insoluble PVPP. Next, the mixture was homogenized at 1000× *g*, while placed in an ice bucket, using a homogenizer (T 25 digital ULTRA-TURRAX^®^, IKA-Werke GmbH and Co. KG, Staufen, Germany) for 2 × 2 min, with a 1 min rest in the middle. After being centrifuged at 4000× *g* for 10 min at 4 °C, the obtained extracts were filtered through Whatman No. 1 filter papers (GE Healthcare, Chalfont Saint Giles, UK) and 0.45-μm cellulose acetate (CA) syringe filters (Test Scientific, Perugia, Italy) and refrigerated at −18 °C until the following analyses.

### 2.5. Determination of PPO Activity

The PPO activity of the extracts was measured according to the method described by Tinello et al. (2018) with some modifications [19]. First, 100 μL of enzyme extract was added to 1 mL of catechol solution (10 mM) as a phenolic substrate previously solubilized in sodium citrate buffer (0.1 M, pH 6.0). An amount of 1 mL of the substrate was used as a blank. The growth trend of the absorbance at 420 nm was recorded at room temperature for 5 min using a spectrophotometer (Varian Carry 50 Bio UV/Vis, Agilent Technologies, Santa Clara, CA, USA). Only the linear part of the curve (Δ absorbance vs. time) was considered for calculating PPO activity, which was then divided with the protein content of the enzyme extract. One arbitrary unit of PPO activity (U/min/µg protein) was defined as the amount of enzyme that produced a 0.001 increase in the absorbance. The protein content of the enzyme extract was determined according to Bradford (1976) [20], using BSA as a protein standard and Coomassie Protein Assay Reagent.

### 2.6. Color Measurements

The colorimetric properties of the eggplants were evaluated using the method described by Dal-Bó and Friere (2022) [21]. The CIELab parameters were measured with an illuminant D65 and observation angle of 10° using a colorimeter (Chroma Meter CR-300, Konica-Minolta, Milan, Italy). The equipment provided the following colorimetric parameters: luminosity (L*) (100: lightness, 0: darkness), a* (+a*: redness, −a*: greenness), and b* (+b*: yellowness, –b*: blueness). The total color difference (ΔE), browning index (BI), hue angle (h*), and chroma (c*) were calculated according to Equations (1)–(4), respectively. For the evaluation of h*, an angle of 180° was assigned as the a* values were negative.

ΔE= [(ΔL*)^2^ + (Δa*)^2^ + (Δb*)^2^]^1/2^ (1)

BI= [100(*x* − 0.31)]/0.172 (2)

h* = 180° + arctan(b*/a*)(3)

c* = (a*^2^ + b*^2^)^1/2^ (4)

where *x*= [(a* + 1.75L*)/(5.645L* + a* − 3.012b*)].

### 2.7. Statistical Analysis

All the data were obtained with at least three replicates and were processed using IBM SPSS Statistics (Version 20.0, SPSS Inc, Chicago, IL, USA). Where necessary, the data were transformed for normalization. After verifying the normal distribution and homogeneity of variance, a one-way analysis of variance (ANOVA) was utilized to investigate the results. Tukey’s honestly significant difference (HSD) test was employed to compare the results. For statistical comparisons between the grafted and not-grafted samples, an independent *t*-test was utilized. The confidence and significance (α) levels were 95% and 0.05, respectively. 

Moreover, Pearson’s correlation was performed between the colorimetric parameters and the PPO activity of the fresh-cut eggplant slices. Origin Pro 2022 (Northampton, MA, USA) was used for graphing the data and performing the principal component analysis (PCA) to determine the correlations between the variables of the extracts from the eggplant fleshes.

## 3. Results and Discussion

### 3.1. PPO Activity

**Figure 2:**
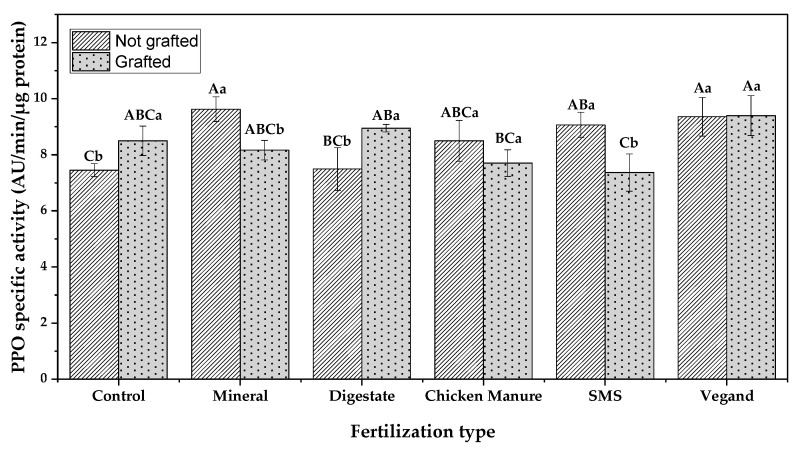
The specific activity of PPO in the fleshes of eggplants. Results are indicated as mean ± SD (*n* = 3). A,B,C: Different capital letters show that there is a significant difference (*p* ≤ 0.05) between the fertilization treatments, according to one-way ANOVA and Tukey’s honestly significant difference (HSD) tests; a,b: Different small letters show that there is a significant difference (*p* ≤ 0.05) between grafted and not-grafted eggplants according to an independent t-test.

Figure 2 shows the specific activity of PPO in the fleshes of eggplants fertilized and grafted using different methods. The results show that, in most cases, grafting could decrease the PPO activity significantly (*p* ≤ 0.05). Among the grafted samples, the eggplants fertilized using chicken manure and SMS had the lowest PPO activity, making them a potential sample with a lower enzymatic browning. In general, fertilization alone significantly increased the PPO activity in all eggplant fleshes (*p* ≤ 0.05), whereas the grafting technique combined with fertilization decreased the PPO activity significantly in most of the samples (*p* ≤ 0.05).

The browning mechanism is explained by the oxidation of plant polyphenols by PPO [22]. It has been proven that fertilization has an impact on the PPO activity and phenolic compounds of plants. As an example, when nitrogen is used as a fertilizer, it can increase the PPO activity in peach fruits [15]. Furthermore, grafting could enhance the apparent quality of fruits and health-related components [17,18]. Another factor affecting enzymatic browning is the type and content of phenolic compounds [23,24,25]. Phenolic compounds are considered to be a substrate for the activity of PPO [9]. These substances are placed inside the vacuoles and bounded by tonoplast. When the senescence of plants is initiated by external stresses, plant cells begin to generate ROS such as superoxide radicals (O_2_^−^), hydrogen peroxide (H_2_O_2_), hydroxyl radicals (^•^OH), and singlet oxygen (^1^O_2_). The generated ROS could react with the membrane lipoproteins, leading to the breakage of the cell membrane, which results in the release of the substrate, phenolic compounds. The subsequent browning reaction happens when the released substrates encounter the PPO and polyphenol peroxidase (POD) enzymes [22].

Sharma et al. (2022) reported that different eggplants had a total phenolic content ranging from 19.3 to 81.1 mg GAE/100 g fresh weight (FW) [1]. The predominant phenolic compound (70–90%) in the eggplant flesh is chlorogenic acid, while other caffeoylquinic acid isomers/derivatives and flavonol (quercetin and myricetin) glycosides are present in low quantities [2]. Liu et al. (2019) investigated the impact of purslane extract on the enzymatic browning of potatoes during processing. They concluded that phenolic compounds, particularly chlorogenic acid, are closely correlated to enzymatic browning [26]. Therefore, the high chlorogenic acid content in eggplants could increase the enzymatic reaction.

A higher content of phenolic compounds in grafted plants when compared to non-grafted plants was reported in several papers. Gisbert et al. (2011) evaluated the impact of grafting using a tomato–eggplant rootstock–scion combination. They proved that phenolic content from grafted (550 mg/kg) eggplants was significantly higher than non-grafted (419 mg/kg) fruits [27]. Moncada et al. (2013) confirmed that grafting has a positive role in improving the phenolic composition of eggplants. However, they concluded that the rootstock has a negligible effect on the phenolic content of fruits [28]. Sabatino et al. (2016) evaluated the effect of the grafting technique on four Sicilian eggplant varieties (Bianca, Sciacca, Marsala, and Sicilia). They reported that grafting could increase the total polyphenol content in three landrace eggplants [29]. This can justify the lower PPO activity in the grafted eggplants in the present study, as higher contents of polyphenols could act as inhibitors of enzymatic browning. 

**Figure 3:**
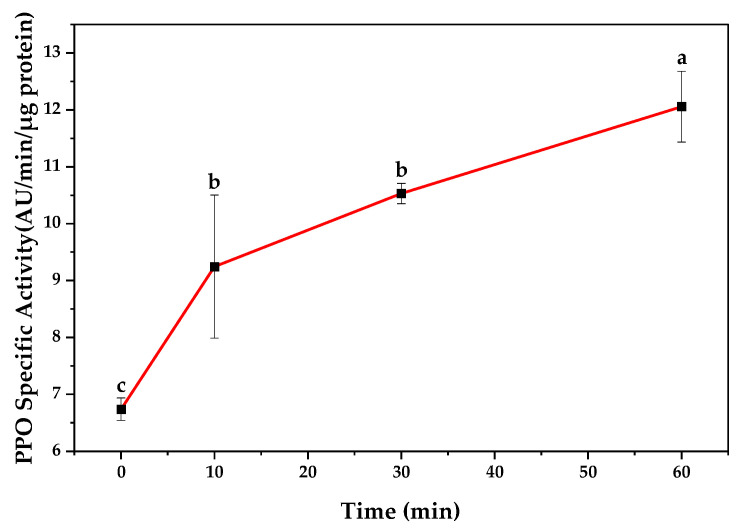
The specific activity of PPO in fresh-cut eggplant slices exposed to room temperature in different times. Results are indicated as mean ± SD (*n* = 3). a,b,c: Different small letters indicate a significant difference (*p* ≤ 0.05, one-way ANOVA, Tukey’s HSD test).

Since the overall pH of eggplants fertilized with chicken manure was lower than the other samples, they were chosen to be assayed for enzymatic browning over time. It is reported that PPO is more effectively inhibited in an acidic environment than in an alkaline one [30]. Moreover, comparing the PPO activity and colorimetric parameters among different treatments, the eggplants fertilized with chicken manure demonstrated a low extent of browning. Figure 3 shows the specific activity of the PPO of fresh-cut eggplant slices fertilized with chicken manure, which were exposed to air over time. As can be seen, there was a significant increase in the activity of the PPO of the eggplant slices over time (*p* ≤ 0.05). The same PPO activity trend was observed in the study carried out by Liu et al. (2021) [9]. The level of phenolic content and the activity of PPO in different cultivars and fruits could lead to a difference in the time of post-cutting browning. The increase in PPO activity and consequent color change could increase ROS generation [1]. Oxidative stress in plants would result in an increase in H_2_O_2_, which is a ROS generated in crops during biotic or abiotic stresses [31]. ROS are produced in the mitochondria through a number of cellular enzymatic and non-enzymatic reactions [32,33]. Liu et al. (2021) confirmed that after fresh cutting, the malondialdehyde (MDA) content, H_2_O_2_, and the rate of O_2_^-^ generation grew over time, which can imbalance the ROS metabolism [9] and lead to a browning reaction. This is consistent with the observations in the present study. Thus, the browning mechanism involves the generation of ROS when there are physical stresses such as cutting in the processing of plant foods [22].

In addition to agronomical treatments, some other methods can contribute to the inactivation of PPO. Sharma et al. (2021) reported that PPO activity increased in eggplants with an increase in their storage period when they were coated with chitosan [34]. Moreover, Lemos et al. (2022) evaluated the effect of UV on fresh-cut eggplants. They reported that the PPO activity in UV-treated eggplants was between 5.98 and 6.38 AU/min/mg protein [35]. These results were lower than the results obtained in the present work, which could be due to the better efficiency of UV in the deactivation of PPO than agronomic treatments.

### 3.2. Colorimetric Parameters

Color is the most prominent quality attribute influencing consumer acceptance of a product [36]. The acceptability of eggplants by consumers varies with the shape, color, and browning intensity of the flesh [34]. The late browning of the fruit flesh plays an important role in the quality of eggplants [37]. It is reported that there is a high correlation between the CIELAB parameters and the content of bioactive compounds in many foods. Thus, evaluating the color properties of foods could be an alternative to the measurement of bioactive compounds owning antioxidant activities. Furthermore, the enzyme activity could also explain color changes in the product, as the color change in eggplants is mainly associated with the PPO enzyme [22,38,39].

Table 1 shows the surface color properties of the fleshes of eggplants fertilized and grafted using different methods, including L*, a*, b*, c*, and h* values. The decrease in the L* value indicates an increase in the degree of discoloration due to browning [40]. There is no significant difference in the L* value among different samples, and all of them had a light color. Similarly, Mozafarian et al. (2021) found that the lightness of the fruit pulp was similar in grafted and non-grafted plants [37].

Moreover, there is no significant difference (*p* > 0.05) among the a* values of the non-grafted samples. However, the a* values in grafted eggplants are significantly different from each other, showing that grafting fertilized eggplants could be effective in inhibiting the enzymatic browning of eggplants. The grafted eggplants fertilized with Vegand had, significantly, the lowest a* values among samples, indicating that they had a greener color. The higher a* values indicate a redder color in the samples.

The c* value could indicate the saturation or color intensity in the samples [21,41]. The results show that there is no significant difference in the intensity of the color among all samples. The colorimetric angle (h*) indicates the final color of the sample, according to the parameters red (0°), yellow (90°), green (180°), and blue (270°) [21]. The values obtained for the fleshes of the eggplants showed angles between approximately 177.74° and 184.60°, indicative of a green coloration.

One of the main problems with eggplants is enzymatic browning on the cut surface, which limits the eggplant’s shelf life [35]. Table 2 shows the color properties of fresh-cut eggplants, fertilized with chicken manure, which were exposed to air over time at room temperature. At room temperature, eggplants clearly exhibit shrinkage, water loss, and discoloration. Sarengaowa et al. (2022) reported that there is an obvious degradation of color and an increase in BI at 25 °C in the center and border of the fresh-cut eggplant samples [40].

**Figure 4:**
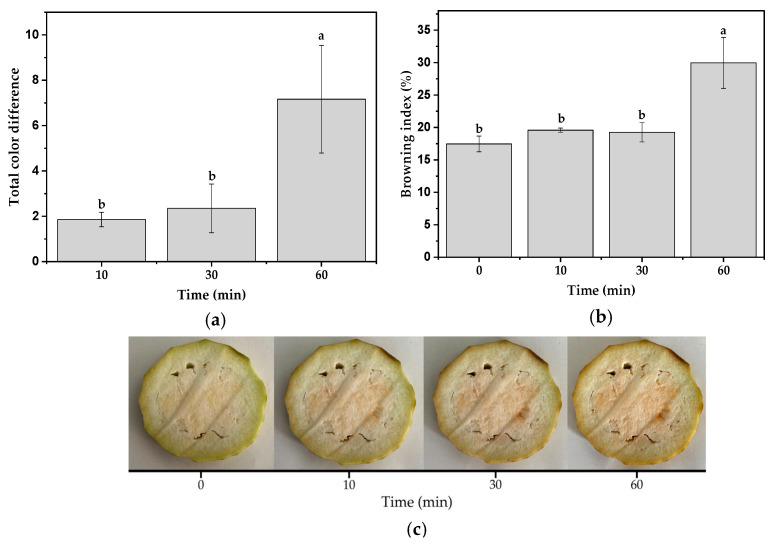
(**a**) The total color difference (ΔE) of samples comparing to time 0 min; (**b**) the browning index (BI) of samples; (**c**) the pictures of the samples in different times. Results are indicated as mean ± SD (*n* = 3). Different small letters indicate a significant difference (*p* ≤ 0.05, one-way ANOVA, Tukey’s HSD test).

Figure 4 shows the total difference of color (ΔE) (a) and the BI (b) of the fresh-cut slices of eggplants which were fertilized with chicken manure and then exposed to air over time. According to the results, there are significant differences in both ΔE and BI after 60 min at room temperature (*p* ≤ 0.05). The pictures of the samples can be seen in Figure 4c. Kacjan Marsic et al. (2014) reported that, after 10 min, the BI was affected by grafting, although after 30 min there were no significant differences [42]. Similarly, Mozafarian et al. (2020) reported that grafting eggplant cv. Madonna reduced the BI of the flesh [43]. However, there are some studies reporting the opposite result [28].

Lemos et al. (2022) evaluated the effect of UV-C treatments on the browning of fresh-cut eggplant. They reported that the BI value increased during storage due to the production of brown polymers stemming from the enzymatic browning that occurs immediately after cutting [35].

### 3.3. Principal Component Analysis (PCA) and Pearson’s Correlation

**Figure 5:**
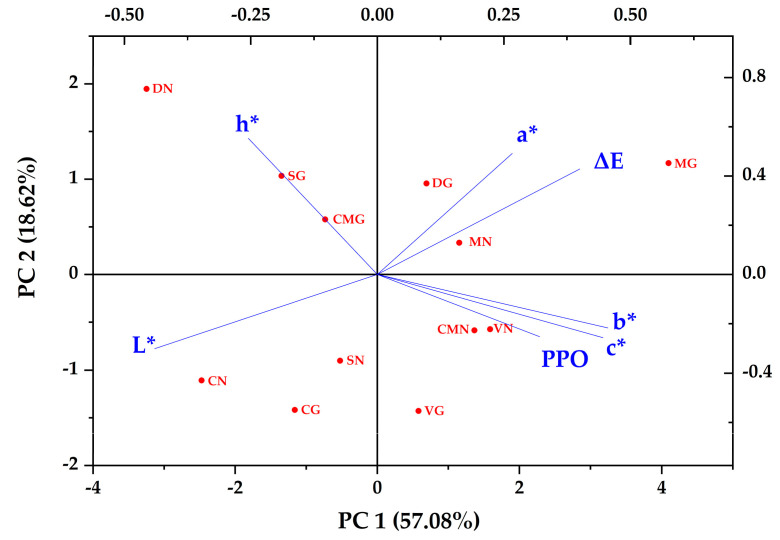
Principal component analysis (PCA) of the colorimetric parameters and PPO activity of eggplant fleshes. Sample Codes are defined in Table 3.

PCA is the most frequently employed clustering method to decide how one sample is different from another [44]. A PCA was performed to indicate the correlation of colorimetric parameters and the PPO activity of the eggplant fleshes cultivated with different types of fertilizers. A total of seven variables were used for the PCA. The biplot of the PCA is shown in Figure 5. The first principal component factor (PC 1, horizontal axis) contributed 57.08% of the total variation, while the second principal component factor (PC 2, vertical axis) described 18.62% of the variations. 

The codes of samples used in PCA are detailed in Table 3. According to the biplot, PPO activity, a*, b*, c*, and ΔE affected PC 1 positively and are on the right side of the plot, showing that they are correlated to each other. However, L* and h* had negative effects on PC 1 and are on the left side of the plot, indicating their negative correlation with PPO activity, a*, b*, c*, and ΔE. These results are consistent with Pearson’s correlation of eggplant slices, excluding that, in the eggplant slices, h* has a positive correlation with the PPO activity, a*, b*, c*, and ΔE. This could be due to the effect of time on the color change of the eggplant slices.

Table 4 shows Pearson’s correlation coefficient among the colorimetric parameters and PPO activity in eggplant slices exposed to air over time. According to the results, there is a significant positive correlation between the PPO activity and a* value. This means that the redder eggplant slices are, the more PPO activity they have. Moreover, the PPO activity has high positive correlations with BI and ΔE, which shows the importance of the PPO enzyme in the color change of the samples.

## 4. Conclusions

The outcomes of this paper could be useful in future research on the effect of agronomic treatments on eggplants, as there is still a need to investigate the ROS profile, phenolic expression, and antioxidant activity of plants cultivated with this methodology. The results of this project highlighted that the PPO activity and colorimetric parameters were mainly affected by the type of fertilizer used. The grafting technique, combined with fertilization, could decrease the PPO activity successfully in most of the samples. This proves that designing functional foods must start from the field. However, it should be recommended to minimize the exposure time of slices to air at room temperature to decrease the waste generated in food production. 

## Figures and Tables

**Table 1:** The colorimetric parameters of eggplant slices exposed to room temperature.

Parameter	Fertilization Type	Grafting	
		No	Yes
L*	Control	81.67 ± 0.31 ^Aa^	81.59 ± 1.08 ^Aa^
	Mineral	80.49 ± 0.92 ^Aa^	80.07 ± 0.88 ^Aa^
	Digestate	81.25 ± 0.56 ^Aa^	80.94 ± 0.61 ^Aa^
	Chicken manure	80.55 ± 0.50 ^Aa^	81.09 ± 0.62 ^Aa^
	SMS	81.21 ± 0.55 ^Aa^	81.05 ± 0.61 ^Aa^
	Vegand	80.49 ± 1.30 ^Aa^	81.09 ± 0.43 ^Aa^
a*	Control	−4.40 ± 0.14 ^Aa^	−4.31 ± 0.58 ^ABa^
	Mineral	−4.0 ± 0.15 ^Aa^	−3.47 ± 0.29 ^Bb^
	Digestate	−4.43 ± 0.70 ^Aa^	−3.57 ± 0.50 ^Ba^
	Chicken manure	−3.81 ± 0.54 ^Aa^	−3.94 ± 0.26 ^ABa^
	SMS	−4.36 ± 0.14 ^Aa^	−3.75 ± 0.38 ^ABa^
	Vegand	−4.30 ± 0.25 ^Aa^	−4.65 ± 0.66 ^Aa^
b*	Control	18.18 ± 1.86 ^Aa^	19.44 ± 1.73 ^Aa^
	Mineral	19.38 ± 1.66 ^Aa^	21.16 ± 2.56 ^Aa^
	Digestate	17.14 ± 0.31 ^Aa^	19.07 ± 2.34 ^Aa^
	Chicken manure	19.53 ± 1.40 ^Aa^	18.35 ± 1.00 ^Aa^
	SMS	18.41 ± 1.35 ^Aa^	17.98 ± 0.52 ^Aa^
	Vegand	19.51 ± 0.43 ^Aa^	19.80 ± 0.70 ^Aa^
c*	Control	18.71 ± 1.83 ^Aa^	19.92 ± 1.70 ^Aa^
	Mineral	19.79 ± 1.61 ^Aa^	21.45 ± 2.52 ^Aa^
	Digestate	17.71 ± 0.26 ^Aa^	19.41 ± 2.21 ^Aa^
	Chicken manure	19.90 ± 1.35 ^Aa^	18.77 ± 0.97 ^Aa^
	SMS	18.93 ± 1.30 ^Aa^	18.37 ± 0.47 ^Aa^
	Vegand	19.98 ± 0.42 ^Aa^	20.34 ± 0.74 ^Aa^
h*	Control	179.24 ± 0.55 ^Aa^	179.88 ± 1.03 ^Aa^
	Mineral	180.23 ± 0.78 ^Aa^	179.61 ± 1.45 ^Aa^
	Digestate	184.60 ± 10.32 ^Aa^	179.99 ± 0.84 ^Aa^
	Chicken manure	177.74 ± 4.78 ^Aa^	179.97 ± 0.47 ^Aa^
	SMS	179.38 ± 0.59 ^Aa^	180.14 ± 0.66 ^Aa^
	Vegand	179.82 ± 0.32 ^Aa^	179.33 ± 1.049 ^Aa^

Results are reported as mean ± SD (*n* = 3). A,B: Different capital letters show that there is a significant difference (*p* ≤ 0.05) between the fertilization treatments, according to one-way ANOVA and Tukey’s HSD tests. a,b: Different small letters show that there is a significant difference (*p* ≤ 0.05) between grafted and not-grafted eggplants, according to the independent *t*-test.

**Table 2:** The colorimetric parameters in the slices of chicken-manure-fertilized eggplant exposed to room temperature for different times.

Parameters	Time (min)			
	0	10	30	60
L*	81.92 ± 0.27 ^a^	80.86 ± 0.94 ^a^	81.36 ± 1.37 ^a^	78.53 ± 2.45 ^a^
a*	−4.04 ± 0.15 ^c^	−2.42 ± 0.18 ^b^	−2.21 ± 0.73 ^ba^	−1.28 ± 0.39 ^a^
b*	16.16 ± 0.78 ^c^	16.57 ± 0.40 ^c^	16.11 ± 1.25 ^c^	21.65 ± 1.66 ^a^
c*	16.66 ± 0.75 ^b^	16.75 ± 0.40 ^b^	16.27 ± 1.29 ^b^	21.69 ± 1.64 ^a^
h*	179.07 ± 0.49 ^a^	175.55 ± 6.13 ^a^	180.06 ± 1.05 ^a^	180.58 ± 3.27 ^a^

Results are reported as mean ± SD (*n* = 3). a,b,c: Different small letters indicate a significant difference (*p* ≤ 0.05, one-way ANOVA, Tukey’s HSD test).

**Table 3:** Code of samples used on principal components analysis (PCA).

Fertilizer	Grafting	Code
-	No	CN
Mineral	No	MN
Digestate	No	DN
Chicken manure	No	CMN
SMS	No	SN
Vegand	No	VN
-	Yes	CG
Mineral	Yes	MG
Digestate	Yes	DG
Chicken manure	Yes	CMG
SMS	Yes	SG
Vegand	Yes	VG

**Table 4:** Pearson’s correlation among different parameters in eggplant slices.

	PPO Activity	L	a*	b*	c*	h*	ΔE
**L**	−0.796						
a*	0.986 *	−0.767					
b*	0.718	−0.979 *	0.717				
c*	0.677	−0.973 *	0.674	0.998 **			
h*	0.386	−0.593	0.239	0.453	0.464		
**ΔE**	0.904	−0.970 *	0.897	0.946	0.927	0.458	
**BI**	0.807	−0.986 *	0.805	0.990 **	0.981 *	0.450	0.982 *

Significant correlation (2-tailed) at * *p* ≤ 0.05, and ** *p* ≤ 0.01.

## Data Availability

Data are contained within the article.
